# Prevalence of conduct problems and social risk factors in ethnically diverse inner-city schools

**DOI:** 10.1186/s12889-021-10834-5

**Published:** 2021-05-03

**Authors:** Rachel Blakey, Craig Morgan, Charlotte Gayer-Anderson, Sam Davis, Stephanie Beards, Seeromanie Harding, Vanessa Pinfold, Kamaldeep Bhui, Gemma Knowles, Essi Viding

**Affiliations:** 1 Health Service and Population Research Department, Institute of Psychiatry, Psychology and Neuroscience, King’s College London, London, UK; 2 ESRC Centre for Society and Mental Health, Institute of Psychiatry, Psychology and Neuroscience, King’s College London, London, SE5 8AF UK; 3 Department of Nutritional Sciences, School of Life Course Sciences, Faculty of Life Sciences & Medicine, King’s College London, Stand, WC2R 2LS UK; 4 The McPin Foundation, 7-14 Great Dover Street, London, SE1 4YR UK; 5 Centre for Psychiatry, Wolfson Institute of Preventive Medicine, Barts & The London, Queen Mary University of London, Charterhouse Square Campus, Old Anatomy Building, London, EC1M 6BQ UK; 6 Developmental Risk and Resilience Unit, University College London, 26 Bedford Way, London, WC1H 0AP UK

**Keywords:** Prevalence, Conduct problems, Adolescence, Ethnicity, Racism, Risk factors, Troublesome friends, Parental bonding, London

## Abstract

**Background:**

In the UK, around 5% of 11–16-year olds experience conduct problems of clinical importance. However, there are limited data on prevalence of conduct problems by ethnic group, and how putative social risk factors may explain any variations in prevalence. This study has two main aims: (1) to estimate the prevalence and nature of conduct problems overall, and by ethnic group and gender, among adolescents in diverse inner-city London schools; (2) to assess the extent to which putative risk factors - racial discrimination, socioeconomic status, parental control, and troublesome friends - explain any observed differences in prevalence of conduct problems between ethnic groups.

**Methods:**

This study uses baseline data from REACH, an accelerated cohort study of adolescent mental health in inner-city London. Self-report questionnaire data were collected on conduct problems and a range of distinct putative social risk factors (including racial discrimination, free school meals, troublesome friends, and parental care and control). A total of 4353 pupils, 51% girls, aged 11–14 participated. We estimated prevalence of conduct problems and used multilevel logistic regression to examine differences by ethnicity and gender and associations with putative risk factors.

**Results:**

Prevalence of conduct problems in inner-city schools was around three times higher than reported in national studies (i.e., 16% [95%CI: 15·2–17·5] vs. 5% [95%CI 4·6–5·9]). Compared with overall prevalence, conduct problems were lower among Indian/Pakistani/Bangladeshi (RR: 0.53 [95% CI:0.31–0.87]) and white British (RR: 0.65 [0.51–0.82]) groups, and higher among black Caribbean (RR: 1.39 [95%CI:1.19–1.62]) and mixed white and black (RR: 1.29 [95% CI: 1.02–1.60]) groups. Risk of conduct problems was higher among those who were exposed to racial discrimination compared with those who were not (RR: 1.95 [95% CI: 1.59–2.31]).

**Conclusions:**

Conduct problems are markedly more common in inner-city schools, and variations in the prevalence of conduct problems are, to some extent, rooted in modifiable social contexts and experiences, such as experiences of racial discrimination.

**Supplementary Information:**

The online version contains supplementary material available at 10.1186/s12889-021-10834-5.

## Background

Conduct problems - aggressive and rule-breaking behaviours - harm individuals, families, and communities, and are a serious public health concern. These behaviours during adolescence are associated with low education attainment and, later, unemployment, criminality, and premature mortality [1, 2].

Conduct problems are common. The 2017 national survey of the Mental Health of Children and Young People in England (MHCYP) estimated around 6% of 11–16 year-olds experience diagnosable conduct problems, and that these are more common among boys (4%) than girls (2%) [3]. While the magnitude of gender differences varies, the estimated risk of conduct problems is consistently higher (50–80%) among boys than girls across large cohort studies [3–5]. However, we have limited information on the extent to which problems vary by region and ethnic group, or gender and ethnic group [3, 6]. Existing studies - although outdated - suggest mixed and black Caribbean adolescents may report symptoms consistent with conduct problems more frequently than their white British peers [6, 7]. If true, this may contribute to long-term ethnic inequalities in health, wellbeing, and mortality.

To address inequality - a persistent social and public health issue - we need to understand the extent and origins of any ethnic disparities. There are numerous risk factors for conduct problems, across multiple domains, including social. Where there are variations in conduct problems by ethnic group, these may be underpinned by differences in exposure to social risks. Candidate social factors that have repeatedly been associated with increased risks of conduct problems include low socio-economic status, high parental control, low parental care, and troublesome peer groups [8–13]. There is evidence that some minority ethnic groups in the UK may be more commonly exposed to these social risks [8, 14]. For example, in 2017 in England, 13% of white British pupils received free school meals (a marker of low socioeconomic status), compared with 23% of black African pupils and 26% of mixed (white and black Caribbean) pupils [15]. In a diverse cohort of adolescents aged 11–13 years, which assessed parental relationships, all minority pupils had lower mean care and higher mean control scores compared with the white group [8]. In addition, given evidence for a greater tendancy to form same-ethnicity friendships than different-ethnicity friendships in adolescence, if there are ethnic inequalities in conduct problems, we might also expect the number of troublesome friends adolescents have to vary by ethnic group [9].

There is also substantial evidence from the US suggesting a causal pathway from experiences of racism to conduct problems [16–18]. Despite this, research on racism and conduct problems in the UK-context is scarce [18]. Evidence from the Determinants of Adolescent Social Well-being and Health (DASH) study, an ethnically diverse cohort from over 15 years ago, suggested high levels of racial discrimination among black Caribbean, mixed ethnicity, and other black and minority ethnic (BAME) groups in south London [14, 19]. Current data on schools in south London (the boroughs of Southwark and Lambeth) also describe this region as being more ethnically diverse, and an area of greater economic inequality compared with UK averages [20–22]. Given that economic deprivation and racism are putative risk factors for conduct problems, that these risks are more commonly experienced by black Caribbean and mixed ethnic groups, and that they are common in the boroughs of Southwark and Lambeth, we may anticipate high levels of conduct problems in these groups, in these inner-city schools.

At present, we have no data on the current distribution of conduct problems or risk factors for conduct problems in inner cities by ethnic group. Given profound social change over the last decade (e.g., increased migration and diversity, prolonged austerity, Brexit, school-based zero-tolerance behaviour policies), it is important to have contemporary estimates of the prevalence of conduct problems and associated risk factors, including by ethnic group, that can inform the development of strategies for prevention and intervention.

This study has two main aims:

1. To estimate the prevalence and nature of conduct problems overall, and by ethnic group and gender, among adolescents in diverse inner-city London schools.
2. To assess the extent to which putative risk factors - racial discrimination, socioeconomic status, parental control, and troublesome friends - explain any observed differences in prevalence of conduct problems between ethnic groups.

To meet these aims, we sought to test the hypothesises that:

1. the prevalence of conduct problems is higher in a) in Southwark and Lambeth (inner-city London) compared with the UK nationally, b) in black Caribbean and mixed ethnic groups compared with white British, and c) among boys than girls.
2. racial discrimination, free school meals (a marker of low SES), higher parental control, lower parental care, and a greater number of troublesome friends are associated with increased risk of conduct problems in all groups
3. these social risks are more common among ethnic groups with the highest prevalence of conduct problems and thereby explain the hypothesised higher prevalence of conduct problems in certain groups

## Method

### Design and setting

Data are from the baseline assessments conducted as part of an accelerated cohort study based in inner-city London boroughs, Southwark and Lambeth (REACH - Resilience, Ethnicity, and Adolescent mental Health) [23]. These boroughs are both ethnically and socio-economically highly diverse [21, 24]_._ We selected 14 state-funded secondary schools based on borough-representative proportions of pupils a) in minority ethnic groups and b) eligible for free school meals. Twelve of these schools participated; one school declined due to time constraints, one school was not included as they agreed after recruitment targets were met. At each participating school, all pupils in school years 7–9 (age 11–14) were eligible to take part (*n*, 4945).

To obtain informed consent, two weeks prior to data collection, researchers delivered school assemblies and distributed information packs to all eligible pupils and their parents/carers. Study information was available via the study website, and where possible, school websites and mailing lists. We asked parents/carers to return a form or contact the school or research team if they did not want their child to participate. Pupils gave written assent before completing the hour-long in-class questionnaire. Researchers were present in all sessions. Baseline questionnaires were administered between February 2016 and January 2018.

### Measures

#### Conduct problems and antisocial behaviours

We assessed conduct problems using the five-item conduct subscale of the youth-self-report Strengths and Difficulties Questionnaire (SDQ) [25]. Items are rated on a three-point scale. Total scores (0–10) were dichotomised at established thresholds: 0–4, “average/slightly raised risk”; 5–10, “very high risk” [26]. Hereafter, scores in the ‘very high risk’ range are referred to as ‘conduct problems’. Confirmatory factor analyses has repeatedly identified the five subscale solution to the SDQ across a number of community and clinical populations [27, 28]. The specificity of the conduct problem subscale is 96%, the negative predictive value is 97%, the sensitivity is 29%, and the positive predictive value is 19% for a DSM-IV diagnosis of conduct disorder. Concurrent validity with the externalising score of the Child Behaviour Checklist is good (r = 0.60) [27]. interrater agreement (*ρ* = 0.30–0.44) and test-retest stability (0.51) of the subscale have also all been found to be satisfactory [27]. In our sample, the internal consistency of the conduct subscale was Cronbach’s α = 0.62.

We measured additional conduct items using a self-report checklist from the development and wellbeing assessment (DAWBA) [29]. The checklist assesses the occurrence (often) of the following behaviours over the previous year: starting fights; bullying/threatening others; running away from home more than once; truancy; stealing; breaking curfew. Response options were “no”, “perhaps”, “definitely”. “Perhaps” and “definitely” were considered positive responses and combined. To align with DSM-IV conduct disorder subdomains, aggressive and non-aggressive rule-breaking, we grouped into (a) fighting and bullying behaviours (often start fights, often bully or threaten others), and (b) other rule-breaking behaviours (running away from home, truancy, stealing, breaking curfew).

#### Socio-demographic factors

Participants selected their ethnic group from 18 categories from the 2011 Office for National Statistics census [30]. Where free text answers under “other” fit extant categories, responses were recoded. Responses “Latino” or equivalent were combined as Latin American. We combined smaller groups and 10 ethnic groups were included in this analysis.

In the UK, during the data collection period, school pupils were eligible for free school meals if their parent/carer was claiming a household means-tested Government benefit [31]. Receipt of free school meals is therefore a marker of low household socioeconomic status. Items on receipt of free school meals, age, school year group, and gender were also included as self-report questions.

#### Putative risk factors

Experiences of discrimination were assessed with the item: “In the past year has anyone made you feel bad or hassled you because of your race, skin colour or where you were born” (yes/no) [32].

Troublesome friends were assessed with the item: “Are many of your friends the sorts of people who often get into trouble for bad behaviour?” Response options were “not at all”, “a few are like that” “many are like that”, or “all are like that”. The latter two options were combined due to small response numbers.

Parental relationships were assessed with the Modified Parental Bonding Instrument (PBI-S) [33, 34]. The 12-items (six on parental care, six on parental control) are rated on a four-point scale (“always = 4”, “never = 0”). Total scores (1–24) were dichotomised: 1–14 “high control”, 15–24 “low control”; 1–8 “high care”, 9–24 “low care” [33, 34]. In this sample, the internal consistency of items in the parental control scale was Cronbach’s α = 0.64, and the parental care scale, Cronbach’s α = 0.88.

### Analysis

We performed analyses in Stata v.15 using weighted data. We developed sample weights using the National Pupil Database (Spring 2016/17 School Census in Lambeth and Southwark).

Analyses were conducted using multilevel logistic regression (melogit and margins commands) with school fitted as a second-level variable. To compare groups, we used the marginalised delta method (nlcom command) to calculate risk ratios (RR) from odds ratios [35].

Without a clear reference category for ethnic group comparisons, we used the overall sample prevalence. We conducted multilevel logistic regressions including ethnic group as a variable and used postestimation command to generate risk estimates for each ethnic group (in these regressions white British was a reference group, see results in Additional File 1). We also conducted each multilevel logistic regression with the relevant covariates and multilevel structure included but no ethnic group variable and used postestimation command to generate a risk estimate for the overall sample. Based on these regressions, we calculated predicted cases and non-cases from each ethnic group and the overall population. The number of predicted cases based on membership of each ethnic group was then compared with the number of predicted cases overall to generate standardised risk ratios. Hence, the estimate of the overall sample prevalence was used as our standard population in each comparison.

To test hypothesis 1a, we used intercept only models to estimate weighted prevalence using imputed data. To test hypothesis 1b, we estimated risk ratios to quantify associations between ethnicity and behavioural outcomes (conduct problems, fighting/bullying, rule-breaking), adjusted for potential confounders (year group, free school meals and gender). To test hypothesis 1c, we estimated associations between gender and behavioural outcomes overall, adjusted for confounders (year group, free school meals and ethnicity), and stratified by ethnic group.

To test hypothesis 2, we estimated risk ratios to quantify associations between putative risk factors (racial discrimination, free school meals, troublesome friends, high parental control, low parental care) and behavioural outcomes adjusted for confounders (year group, ethnicity, gender).

To test hypothesis 3, we first estimated risk ratios to quantify associations between ethnicity and each putative risk factor adjusted for confounders (year group, free school meals, gender) and then added each risk factor sequentially to a base model with ethnic group in to assess whether these factors explained variations by ethnic group. We tested improvement in model fit using likelihood ratio tests. All variables that improved model fit, at *p* < 0·05, were retained in the model.

### Sensitivity analyses

Measurers of antisocial behaviours, troublesome friends, and parental relationships were not administered at two (originally pilot, *n* = 818) schools. To assess any impact of pilot school data on magnitude of estimates or associations, we repeated analyses of conduct problems and racial discrimination excluding pilot school data.

### Missing data

To account for bias due to item non-response we carried out multi-level multiple imputation in REALCOM [36]. We performed imputation on two data sets, one including pilot schools *n* = 4353 and one excluding pilot schools *n* = 3535. In each imputation model, we included all available variables used in analyses with school fitted as a second-level variable. 20 imputations were used.

## Results

### Participants

**Fig. 1:**
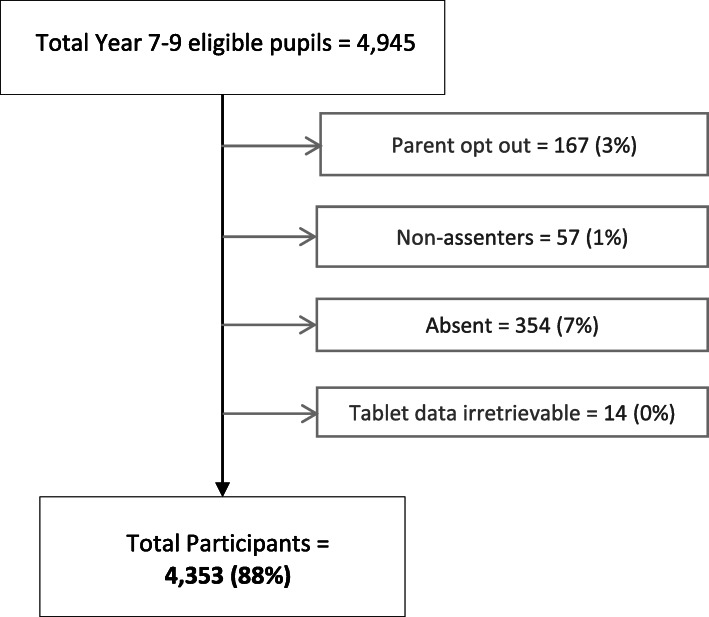
Participation Rate Flow Diagram

A total of 4353 pupils (51% girls, 85% minority ethnic groups) participated (Fig. 1). The mean age was 12·4(SD 0·96) years old. Sample demographics are presented in Table 1. For demographics stratified by gender, see Additional File 2.

**Table 1:** Descriptive Data

	Conduct problems				* Fighting and Bullying				*Rule-breaking			
	Low risk	High risk	Missing	χ2	No	Perhaps/Definitely	Missing	χ2	No	Perhaps/Definitely	Missing	χ2
	***n (%)***	***n (%)***	***n (%)***		***n (%)***	***n (%)***	***n (%)***		***n (%)***	***n (%)***	***n (%)***	
Total Sample (4353)	3572 (82)	710 (16)	71 (2)		2186 (62)	1132 (32)	217 (6)		2036 (58)	1240 (35)	259 (7)	
Gender				6.13, *p* = 0.013				0·10, p = 0·752				8·34, *p* = 0·004
Boys	1720 (80)	378 (18)	40 (2)		1034 (60)	542 (32)	134 (8)		925 (54)	628 (37)	157 (9)	
Girls	1852 (84)	332 (15)	31 (1)		1152 (63)	590 (33)	83 (5)		1111 (61)	612 (34)	102 (6)	
Year group				0.07, *p* = 0·968				3·04, *p* = 0·219				53·40, *p* < 0·001
7	1308 (82)	263 (17)	22 (1)		836 (63)	398 (30)	95 (7)		842 (63)	368 (28)	119 (9)	
8	1163 (82)	228 (16)	30 (2)		685 (60)	372 (33)	78 (7)		642 (57)	414 (36)	79 (7)	
9	1101 (82)	219 (16)	19 (1)		665 (62)	362 (34)	44 (4)		552 (52)	458 (43)	61 (6)	
Ethnicity				60.80, *p* < 0·001				54·51, *p* < 0·001				59·63, *p* < 0·001
Black African	907 (81)	185 (17)	21 (2)		523 (56)	335 (36)	68 (7)		515 (56)	321 (35)	90 (10)	
Black Caribbean	540 (75)	159 (22)	20 (3)		292 (58)	166 (33)	45 (9)		254 (51)	202 (40)	47 (9)	
Indian, Pakistani, Bangladeshi	165 (91)	14 (8)	2 (1)		106 (69)	39 (25)	8 (5)		115 (75)	31 (20)	7 (5)	
Latin American	180 (83)	35 (16)	2 (1)		75 (50)	68 (45)	8 (5)		69 (46)	62 (41)	20 (13)	
Mixed white and black	296 (78)	81 (21)	3 (1)		191 (62)	99 (32)	18 (6)		164 (53)	125 (41)	19 (6)	
Other black	101 (80)	25 (20)	1 (1)		57 (53)	44 (41)	6 (6)		56 (52)	44 (41)	7 (7)	
Other mixed/multiple	195 (82)	39 (16)	3 (1)		129 (62)	66 (32)	14 (7)		107 (51)	90 (43)	12 (6)	
Other White	345 (84)	59 (14)	5 (1)		195 (64)	92 (30)	16 (5)		178 (59)	107 (35)	18 (6)	
Other/unknown	245 (81)	49 (16)	9 (3)		155 (63)	71 (29)	19 (8)		149 (61)	78 (32)	18 (7)	
White British	598 (90)	64 (10)	5 (1)		463 (73)	152 (24)	15 (2)		429 (68)	180 (29)	21 (3)	
Putative risk factors												
Receives free school meals												
yes	762 (78)	197 (20)	17 (2)	15.41, *p* < 0·001	391 (64)	224 (36)	42 (6)	1·82, *p* = 0·177	354 (54)	253 (39)	50 (8)	4·48, *p* = 0·034
No	2629 (84)	470 (15)	38 (1)		1672 (66)	844 (34)	150 (6)		1566 (59)	921 (35)	179 (7)	
Experienced Racial Discrimination												
yes	805 (74)	272 (25)	4 (0)	86.45, *p* < 0·001	432 (49)	422 (48)	32 (4)	127·75, *p* < 0·001	418 (47)	422 (48)	46 (5)	81·82, *p* < 0·001
No	2484 (86)	370 (13)	31 (1)		1651 (70)	641 (27)	58 (3)		1532 (65)	740 (31)	78 (3)	
*Troublesome friends				211.62, *p* < 0·001				276·17, *p* < 0·001				282·18, *p* < 0·001
None	956 (91)	79 (8)	14 (1)		805 (77)	204 (19)	40 (4)		796 (76)	203 (19)	50 (5)	
A few	1613 (84)	285 (15)	12 (1)		1219 (64)	648 (34)	43 (2)		1088 (57)	757 (40)	65 (3)	
Many or all	237 (60)	151 (38)	7 (2)		122 (31)	255 (64)	18 (5)		117 (30)	250 (63)	28 (7)	
*Parental Control												
*High*	1006 (80)	240 (19)	14 (1)	26.01, *p* < 0·001	750 (60)	466 (37)	44 (3)	22·69, *p* < 0·001	702 (56)	495 (39)	63 (5)	14·90, *p* < 0·001
*Low*	1261 (87)	174 (12)	10 (1)		1002 (69)	420 (29)	23 (2)		933 (65)	481 (33)	31 (2)	
*Parental Care								52·10, *p* < 0·001				99·68, *p* < 0·001
High	1253 (89)	144 (10)	15 (1)	57.41, *p* < 0·001	1002 (71)	374 (26)	36 (3)		978 (69)	385 (27)	49 (3)	
Low	1016 (79)	268 (21)	10 (1)		752 (58)	511 (39)	31 (2)		660 (51)	589 (46)	45 (3)	

Complete cases reported, percentages and n unweighted

*Administered at 10 of 12 schools, total sample (*n* = 3535)

Around 16% (95% CI: 15·2–17·5) scored above the threshold for conduct problems. This is over three times higher than the estimated national prevalence of conduct problems using the same measure in the UK Millennium Cohort (MCS) at age 14 (5·2% [95%CI 4·6–5·9]) (personal communication), and the MHCYP^3^ age 11–16 (6.2% [CI’s not available]) (Hypothesis 1a). Overall, an estimated 35·4% (95% CI: 31·2–39·5) reported often starting fights and/or bullying or threatening people and 38·7% (95% CI 34·9–42·6) reported engaging in other rule-breaking behaviours in the last year (estimates reported on weighted and imputed data).

### Conduct problems by ethnicity

There was variation by ethnic group in prevalence of conduct problems (Hypothesis 1b; Table 2). Generally, conduct problems were less common among Indian/Pakistani/Bangladeshi (8·8% [95% CI: 3·0–14·5]), and white British (10·6% [95% CI: 7·9–13·3]) groups and more common among black Caribbean (23·2% [95%CI: 19·6–26·9]) and mixed white and black ethnic groups (21·9% [95%CI: 17·5–26·3]). The prevalence was close to the overall estimate among black African (16·4% [95%CI:13·4–19·5]), Latin American (15·6% [95%CI: 10·3–21·0]), and other mixed groups (17·4% [95% CI: 13·7–21·2]). It follows that the prevalence in all other ethnic groups was higher than in Indian/Pakistani/Bangladeshi and white British groups. Indeed, compared with overall prevalence, all behavioural problems were less common among white British adolescents, e.g., conduct problems RR 0·65 (95% CI 0·51–0·82), fighting/bullying RR 0·71 (95% CI 0·60–0·83), and rule-breaking RR 0·80 (95% CI 0·69–0·91). Among black Caribbean adolescents, there was a higher prevalence of conduct problems (RR 1·39 [95% CI 1·19–1·62]), rule-breaking behaviours (RR 1·16 [95% CI 1·02–1·32]), and, to a lesser extent, fighting/bullying behaviours (RR 1·10 [95% CI 0·95–1·26]). Among the Latin American group, fighting/bullying were more common (RR 1·33 [95% CI 1·03–1·67]), but conduct problems were not (RR 0·97 [95%CI 0·68–1·35]).

**Table 2:** Risk Ratios for conduct problems by ethnic group

	Conduct problems		*Fighting/Bullying		*Rule-breaking	
	Unadjusted RR (95%CI)	Adjusted RR (95%CI)	Unadjusted RR (95%CI)	Adjusted RR (95%CI)	Unadjusted RR (95%CI)	Adjusted RR (95%CI)
Ethnicity						
Black African	0.99 (0.88–1.10)	0.99 (0.85–1.14)	1.10 (0.98–1.21)	1.10 (0.99–1.21)	0.98 (0.88–1.09)	0.98 (0.88–1.09)
Black Caribbean	1.40 (1.18–1.50)	1.39 (1.19–1.62)	1.11 (0.96–1.27)	1.10 (0.95–1.26)	1.18 (1.03–1.34)	1.16 (1.02–1.32)
Indian/Pakistani/Bangladeshi	0.53 (0.47–0.95)	0.53 (0.31–0.87)	0.89 (0.65–1.18)	0.90 (0.67–1.19)	0.61 (0.43–0.84)	0.62 (0.44–0.86)
Latin American	0.95 (0.76–1.27)	0.97 (0.68–1.35)	1.33 (1.04–1.68)	1.33 (1.03–1.67)	1.25 (0.98–1.57)	1.22 (0.96–1.54)
Mixed white and black	1.32 (1.07–1.51)	1.29 (1.02–1.60)	1.01 (0.83–1.22)	1.00 (0.82–1.20)	1.13 (0.95–1.34)	1.13 (0.94–1.33)
Other black	1.24 (0.91–1.67)	1.24 (0.81–1.81)	1.29 (0.96–1.71)	1.29 (0.95–1.71)	1.21 (0.90–1.59)	1.22 (0.91–1.61)
Other mixed/multiple	1.05 (0.85–1.36)	1.05 (0.75–1.42)	1.03 (0.81–1.29)	1.03 (0.81–1.28)	1.25 (1.02–1.52)	1.25 (1.02–1.52)
Other white	0.87 (0.68–1.03)	0.89 (0.68–1.14)	0.91 (0.74–1.11)	0.91 (0.74–1.11)	0.98 (0.81–1.18)	0.99 (0.82–1.19)
Other/unknown	1.02 (0.76–1.18)	1.00 (0.74–1.31)	0.91 (0.72–1.14)	0.91 (0.72–1.13)	0.90 (0.72–1.11)	0.88 (0.70–1.09)
White British	0.64 (0.61–0.86)	0.65 (0.51–0.82)	0.70 (0.60–0.82)	0.71 (0.60–0.83)	0.79 (0.68–0.91)	0.80 (0.69–0.91)

*RR* Risk ratio, *95%CI* 95% Confidence Interval

Reference group is overall prevalence

Adjusted for clustering by school, year group, gender, and free school meals

*Administered at 10 of 12 schools, total sample (n = 3535).

### Conduct problems by gender within ethnic groups

Compared with boys, prevalence of conduct problems (RR 0·81 [95% CI 0·67–0·95]) and rule-breaking behaviours (RR 0·85 [95% CI 0·71–1·00]) were lower among girls (Hypothesis 1c). There was no evidence of a gender difference in fighting/bullying (RR 1·02 [95% CI 0·84–1·19]).

There were notable variations by ethnic group in gender differences. There was a lower prevalence of conduct problems among girls in the other white (RR 0·38 [95%CI 0·10–0·65]), and Latin American (RR 0.38 [95%CI 0.12–0.64]) groups. However, there were no differences in black African and black Caribbean groups (Table 3). Similarly, there were gender differences in rule-breaking in some groups (e.g., white British (RR 0·66 [95% CI 0·46–0·86]), other mixed (RR 0.58 [95% CI 0·41–0·76]), but not others. There was no evidence of a gender difference in risks of fighting/bullying within any ethnic group – estimates were high for girls and boys.

**Table 3:** Risk Ratios for conduct problems by gender within ethnic group

	Conduct problems		*Fighting/Bullying		*Rule-breaking	
	Unadjusted RR (95%CI)	Adjusted RR (95%CI)	Unadjusted RR (95%CI)	Adjusted RR (95%CI)	Unadjusted RR (95%CI)	Adjusted RR (95%CI)
Overall gender difference						
Girl	0.83 (0.70–0.96)	0.81 (0.67–0.95)	1·02 (0·87–1·17)	1·02 (0·84–1·19)	0·89 (0·76–1·02)	0·85 (0·71–1·00)
Within ethnic group						
Black African	0.98 (0.77–1.18)	0.98 (0.77–1.18)	0.97 (0.83–1.11)	0.97 (0.82–1.13)	0.98 (0.78–1.18)	0.97 (0.76–1.19)
Black Caribbean	0.99 (0.82–1.16)	1.01 (0.76–1.27)	0.86 (0.65–1.07)	0.87 (0.63–1.11)	0.83 (0.64–1.02)	0.80 (0.59–1.00)
Indian/Pakistani/Bangladeshi	1.31 (0.22–2.41)	1.76 (− 0.10–3.61)	0.93 (0.07–1.80)	0.89 (0.10–1.68)	0.78 (0.12–1.43)	0.74 (0.03–1.45)
Latin American	0.37 (0.11–0.63)	0.38 (0.12–0.64)	1.16 (0.62–1.69)	0.99 (0.52–1.45)	1.01 (0.51–1.50)	0.99 (0.52–1.45)
Mixed white and black	0.78 (0.54–1.02)	0.78 (0.54–1.02)	0.90 (0.55–1.26)	0.89 (0.53–1.26)	1.08 (0.81–1.36)	1.13 (0.76–1.49)
Other black	0.71 (0.21–1.21)	0.72 (0.23–1.21)	0.98 (0.61–1.34)	0.96 (0.58–1.33)	0.88 (0.59–1.16)	0.85 (0.50–1.20)
Other mixed/multiple	1.28 (0.62–1.95)	1.23 (0.53–1.93)	1.07 (0.60–1.54)	1.05 (0.52–1.58)	0.66 (0.47–0.86)	0.58 (0.41–0.76)
Other white	0.40 (0.11–0.68)	0.38 (0.10–0.65)	1.30 (0.78–1.81)	1.33 (0.81–1.85)	0.97 (0.81–1.13)	0.94 (0.74–1.15)
Other/unknown	0.94 (0.26–1.62)	0.93 (0.27–1.58)	0.98 (0.45–1.52)	1.01 (0.42–1.59)	0.85 (0.47–1.22)	0.82 (0.39–1.24)
White British	0.80 (0.35–1.26)	0.77 (0.27–1.26)	1.02 (0.75–1.29)	1.02 (0.76–1.28)	0.70 (0.52–0.87)	0.66 (0.46–0.86)

*RR* Risk ratio*, 95%CI* 95% Confidence Interval

For gender comparisons, boys are used as the reference. For ethnic comparisons, the overall sample prevalence is used as the reference

Adjusted for clustering by school, year group, and free school meal

*Administered at 10 of 12 schools, total sample (*n* = 3535)

### Putative risk factors

Each putative risk factor – racial discrimination, free school meals, troublesome friends, and parental care and control – was associated with increased risk of each outcome (Hypothesis 2; Table 4). For example, among those who experienced racial discrimination the risk of conduct problems was two times greater than among those who had not (RR 1·95 [95%CI 1·59–2·31]). Among those who reported many or all their friends got into trouble, the risk of conduct problems was around 5 times greater than for those who reported none of their friends got into trouble (RR 4.80 [95% CI 3.75–5.85]).

**Table 4:** Risk Ratios for conduct problems by putative risk factors

	Conduct problems		*Fighting/Bullying		*Rule-breaking	
	Unadjusted RR (95%CI)	Adjusted RR (95%CI)	Unadjusted RR (95%CI)	Adjusted RR (95%CI)	Unadjusted RR (95%CI)	Adjusted RR (95%CI)
Receives free school meals	1.35 (1.05–1.65)	1.32 (1.01–1.63)	1.09 (0.93–1.25)	1.09 (0.89–1.30)	1.12 (0.98–1.26)	1.14 (0.95–1.33)
Experienced Racial Discrimination	1.88 (1.51–2.25)	1.95 (1.59–2.31)	1.70 (1.52–1.88)	1.84 (1.59–2.08)	1.50 (1.37–1.63)	1.62 (1.44–1.80)
*A few troublesome friends	1.91 (1.57–2.26)	1.82 (1.47–2.16)	1.68 (1.40–1.96)	1.82 (1.46–2.18)	1.91 (1.67–2.14)	2.09 (1.86–2.31)
*Many or all troublesome friends	4.81 (3.68–5.94)	4.80 (3.75–5.85)	3.13 (2.63–3.64)	4.00 (3.24–4.76)	3.13 (2.70–3.57)	3.99 (3.29–4.69)
*Parental High Control	1.49 (1.23–1.75)	1.48 (1.21–1.75)	1.25 (1.12–1.38)	1.26 (1.07–1.44)	1.17 (1.03–1.30)	1.21 (1.05–1.37)
*Parental Low Care	1.89 (1.52–2.26)	1.99 (1.59–2.40)	1.44 (1.27–1.60)	1.52 (1.33–1.72)	1.58 (1.41–1.76)	1.69 (1.45–1.92)

*RR* Risk ratio*, 95%CI* 95% Confidence Interval

Adjusted for clustering by school, year group, gender, and free school meals (except where ‘free school meals’ is the independent variable)

*Administered at 10 of 12 schools, total sample (*n* = 3535)

### Putative risk factors by ethnicity

The occurrence of putative risk factors was largely similar among most ethnic groups included (Hypothesis 3; Table 5). The white British group was the exception. Every putative risk factor (except parental low care) was less common among the white British group. For instance, exposure to discrimination was similar among most ethnic groups including Latin American, black Caribbean, black African, other black, and mixed white and black. By contrast, exposure to racial discrimination was lower for the white British group (17% vs. 28% overall), (RR 0·60 [95% CI 0·49–0·72]). There was also some evidence of putative risks being more common in the mixed/multiple, mixed white and black groups, and the black Caribbean groups. Indeed, racial discrimination was most common among the mixed/multiple group relative to the overall sample. (37% vs 28% overall), (RR 1.36 [95% CI 1.07–1.69]). The mixed white and black group were more likely to receive free school melas than the overall sample (33% vs 24% overall), (RR 1.36 [95% CI 1.13–1.62]), and the black Caribbean group were more likely to have a few or many troublesome friends (76% vs. 70% overall), (RR 1·10 [95% CI 0·99–1·21]).

**Table 5:** Risk ratios for putative risk factors by ethnic group

	Receives free school meals	Experienced Racial Discrimination	*A few to many or all troublesome friend’s vs none	*Parental High Control	*Parental Low Care
	% (95%CI)	% (95%CI)	% (95%CI)	% (95%CI)	% (95%CI)
Overall prevalence in sample	0.24 (0.17–0.30)	0.28 (0.24–0.32)	0.70 (0.66–0.74)	0.47 (0.43–0.52)	0.48 (0.45–0.50)
Ethnicity	RR (95%CI)	RR (95%CI)	RR (95%CI)	RR (95%CI)	RR (95%CI)
Black African	1.04 (0.92–1.17)	1.10 (0.98–1.24)	1.03 (0.95–1.11)	1.12 (1.02–1.22)	1.06 (0.96–1.16)
Black Caribbean	1.12 (0.96–1.29)	0.96 (0.80–1.13)	1.10 (0.99–1.21)	1.13 (1.00–1.28)	1.00 (0.88–1.14)
Indian/Pakistani/Bangladeshi	0.82 (0.57–1.14)	1.18 (0.88–1.55)	0.95 (0.77–1.15)	1.12 (0.89–1.39)	0.89 (0.69–1.14)
Latin American	0.72 (0.51–0.99)	0.98 (0.71–1.33)	0.97 (0.79–1.17)	1.27 (1.02–1.56)	1.14 (0.91–1.42)
Mixed white and black	1.36 (1.13–1.62)	1.00 (0.80–1.23)	1.09 (0.96–1.24)	0.97 (0.82–1.15)	1.00 (0.84–1.17)
Other black	0.93 (0.62–1.35)	0.96 (0.64–1.38)	0.98 (0.77–1.23)	1.05 (0.78–1.37)	0.87 (0.63–1.16)
Other mixed/multiple	1.16 (0.90–1.48)	1.36 (1.07–1.69)	0.97 (0.82–1.15)	0.92 (0.74–1.13)	1.03 (0.84–1.25)
Other white	0.68 (0.53–0.87)	0.79 (0.62–1.01)	0.95 (0.82–1.09)	0.86 (0.72–1.03)	0.87 (0.72–1.03)
Other/unknown	1.19 (0.95–1.47)	1.17 (0.93–1.45)	0.97 (0.83–1.13)	1.05 (0.87–1.25)	1.05 (0.87–1.25)
White British	0.86 (0.72–1.01)	0.60 (0.49–0.72)	0.90 (0.82–1.00)	0.65 (0.56–0.75)	0.98 (0.87–1.09)

*RR* Risk ratio*, 95%CI* 95% Confidence Interval

Reference group is the overall prevalence

Adjusted for clustering by school

*Administered at 10 of 12 schools, total sample (*n* = 3535).

We next considered whether these risk factors might explain observed variations in conduct problems by ethnic group. When we added these putative explanatory factors, the magnitude of variations in prevalence by ethnic group generally remained similar (Table 6). However, for the white British group, when models were adjusted, the estimated RR approached 1, i.e. no difference in risk between white British adolescents and the overall prevalence. This suggests these factors account for some of the disparity in conduct problems and antisocial behaviours between the white British group and the overall prevalence. For example, in the base model (adjusted for age and free school meals) there was strong evidence that the risk of conduct problems for white British adolescents was lower than the overall sample (RR 0·65 [95%CI 0·50–0·82]). In Model 4 (adjusted for racial discrimination, troublesome friends, parental control and care) there was weaker evidence for a difference in risk of conduct problems among white British adolescents compared with the overall sample (RR 0·81 [95%CI 0·64–1·01]). This pattern may reflect attenuation of cumulative advantage of white British adolescents (who are exposed to fewer risk factors for conduct problems than the overall sample) as we adjust the model. In addition, there was some evidence of attenuated risks of conduct problems as models were adjusted in those groups who were more commonly exposed to putative risk factors. For example, in the base model the black Caribbean group were at elevated risk of conduct problems compared with the overall sample (RR 1·37 [95%CI 1·12–1·64]) and this estimate was reduced in model 3 through adjusting for racial discrimination, friends who get into trouble, and high parental control (RR 1.25 [95%CI 1.02–1.52]).

**Table 6:** Risk ratios for conduct problems by ethnic group accounting for putative risk factors

	Base model: age, gender, free school meals	Model 1: base model + racial discrimination	Model 2: model 1+ friends who get into trouble	Model 3: model 2 + high control	Model 4: model 3 + low perceived parental care
	RR (95%CI)	RR (95%CI)	RR (95%CI)	RR (95%CI)	RR (95%CI)
*Conduct problems*					
Black African	1.00 (0.84–1.17)	0.98 (0.83–1.15)	0.96 (0.81–1.13)	0.95 (0.80–1.12)	0.93 (0.78–1.10)
Black Caribbean	1.37 (1.12–1.64)	1.35 (1.11–1.63)	1.28 (1.04–1.55)	1.25 (1.02–1.52)	1.27 (1.03–1.54)
Indian/Pakistani/Bangladeshi	0.49 (0.25–0.85)	0.45 (0.22–0.80)	0.49 (0.26–0.86)	0.50 (0.26–0.86)	0.50 (0.26–0.86)
Latin American	1.07 (0.70–1.56)	1.03 (0.67–1.53)	1.04 (0.68–1.54)	1.00 (0.64–1.49)	0.96 (0.61–1.44)
Mixed white and black	1.25 (0.95–1.60)	1.24 (0.95–1.59)	1.21 (0.92–1.56)	1.23 (0.94–1.58)	1.23 (0.94–1.58)
Other black	1.33 (0.84–1.99)	1.34 (0.85–2.01)	1.36 (0.86–2.03)	1.36 (0.86–2.03)	1.42 (0.91–2.11)
Other mixed/multiple	1.01 (0.70–1.41)	0.96 (0.65–1.35)	0.97 (0.66–1.36)	0.97 (0.66–1.36)	0.94 (0.64–1.33)
Other white	0.86 (0.62–1.16)	0.91 (0.66–1.22)	0.92 (0.67–1.23)	0.94 (0.68–1.25)	0.98 (0.72–1.30)
Other/unknown	1.09 (0.79–1.46)	1.05 (0.75–1.42)	1.08 (0.78–1.46)	1.06 (0.76–1.43)	1.06 (0.76–1.43)
White British	0.65 (0.50–0.82)	0.71 (0.56–0.90)	0.77 (0.61–0.96)	0.80 (0.64–1.00)	0.81 (0.64–1.01)
*Fighting/Bullying*					
Black African	1.10 (0.99–1.21)	1.08 (0.97–1.20)	1.06 (0.95–1.18)	1.06 (0.95–1.18)	1.05 (0.94–1.17)
Black Caribbean	1.10 (0.95–1.26)	1.09 (0.94–1.25)	1.03 (0.88–1.19)	1.02 (0.88–1.18)	1.02 (0.88–1.18)
Indian/Pakistani/Bangladeshi	0.90 (0.67–1.19)	0.87 (0.64–1.16)	0.91 (0.68–1.21)	0.91 (0.68–1.21)	0.92 (0.68–1.21)
Latin American	1.33 (1.03–1.67)	1.31 (1.02–1.66)	1.30 (1.01–1.65)	1.29 (1.00–1.63)	1.27 (0.98–1.61)
Mixed white and black	1.00 (0.82–1.20)	0.99 (0.82–1.20)	0.97 (0.80–1.18)	0.97 (0.80–1.18)	0.97 (0.80–1.18)
Other black	1.29 (0.95–1.71)	1.30 (0.96–1.72)	1.31 (0.97–1.73)	1.31 (0.97–1.73)	1.34 (0.99–1.76)
Other mixed/multiple	1.03 (0.81–1.28)	0.98 (0.76–1.23)	0.98 (0.77–1.24)	0.98 (0.77–1.24)	0.98 (0.77–1.24)
Other white	0.91 (0.74–1.11)	0.95 (0.77–1.15)	0.97 (0.79–1.18)	0.98 (0.80–1.19)	0.99 (0.81–1.20)
Other/unknown	0.91 (0.72–1.13)	0.88 (0.69–1.10)	0.91 (0.72–1.13)	0.91 (0.72–1.13)	0.90 (0.71–1.12)
White British	0.71 (0.60–0.83)	0.76 (0.65–0.88)	0.82 (0.70–0.94)	0.83 (0.72–0.96)	0.83 (0.72–0.96)
*Rule-breaking*					
Black African	0.98 (0.88–1.09)	0.97 (0.87–1.07)	0.95 (0.86–1.06)	0.95 (0.85–1.05)	0.94 (0.84–1.05)
Black Caribbean	1.16 (1.02–1.32)	1.16 (1.01–1.32)	1.10 (0.96–1.26)	1.09 (0.95–1.25)	1.10 (0.96–1.26)
Indian/Pakistani/Bangladeshi	0.62 (0.44–0.86)	0.59 (0.41–0.82)	0.63 (0.44–0.86)	0.63 (0.44–0.86)	0.64 (0.45–0.88)
Latin American	1.22 (0.96–1.54)	1.21 (0.95–1.53)	1.21 (0.95–1.53)	1.20 (0.93–1.51)	1.18 (0.92–1.49)
Mixed white and black	1.13 (0.94–1.33)	1.12 (0.94–1.33)	1.09 (0.91–1.29)	1.09 (0.91–1.29)	1.09 (0.91–1.29)
Other black	1.22 (0.91–1.61)	1.25 (0.93–1.64)	1.26 (0.94–1.65)	1.26 (0.94–1.65)	1.26 (0.94–1.65)
Other mixed/multiple	1.25 (1.02–1.52)	1.21 (0.98–1.47)	1.22 (0.99–1.49)	1.22 (0.99–1.49)	1.22 (0.99–1.49)
Other white	0.99 (0.82–1.19)	1.02 (0.84–1.22)	1.04 (0.86–1.24)	1.04 (0.86–1.24)	1.07 (0.89–1.27)
Other/unknown	0.88 (0.70–1.09)	0.86 (0.69–1.07)	0.90 (0.72–1.11)	0.89 (0.71–1.10)	0.88 (0.70–1.08)
White British	0.80 (0.69–0.91)	0.84 (0.73–0.96)	0.89 (0.78–1.02)	0.91 (0.79–1.03)	0.91 (0.79–1.04)

*RR* Risk ratio*, 95%CI* 95% Confidence Interval

Reference group is the overall prevalence in each model

For the purpose of comparison across models, all models include observations from 10 schools *n* = 3535

### Sensitivity analyses

The inclusion of two pilot schools in analyses of conduct problems and racial discrimination did not substantively alter any of the reported associations [see sensitivity analyses in Additional File 3].

## Discussion

Our analyses produced several notable findings. First, adolescent conduct problems were three-times higher in our inner-city sample than in recent national samples (16% vs 5%) [3, 28]. Second, conduct problems were more common among boys compared with girls in some, but not all (e.g., black, and mixed groups), ethnic groups. Third, the white British group differed from the overall sample, with lower risks of conduct problems and antisocial behaviours. Fourth, these disparities were largely explained by variations in social risk factors. Fifth, across all ethnic groups, racial discrimination, free school meals, parental control, parental care, and friendships with peers who get into trouble were all associated with conduct problems and antisocial behaviours. Finally, white British adolescents were less commonly exposed to each social risk.

### Limitations

Several methodological limitations should be considered before interpreting our findings. For example, not all pupils participated, which may have biased the sample. The primary reason for non-participation was absence from school and, as conduct problems are associated with school absence, our sample may underestimate prevalence. Conversely, self-report single-informant SDQ scores, while sensitive to true negatives (95%), tend to be over-inclusive [25] and may overestimate conduct problems. The relative impact of each potential bias is unclear. Still, it seems likely that conduct problems are substantially more common in inner-city schools given the high threshold we used for conduct problems, and the comparison with MCS prevalence estimates using the same self-report measure at the same threshold.

Our self-report measure of conduct problems may not reflect the same construct for different ethnic groups. Conduct problems are a multifaceted and heterogeneous group of behaviours which may present differently across ethnic groups. Moreover, self-report measures are susceptible to information bias. Adolescents may diminish or exaggerate behaviours according to social acceptability or desirability, which may vary by cultural background. This noted, a strength of self-report measures, compared with teacher-report, is that they are not (directly) impacted by implicit gendered or racial biases from teachers. Still, the potential for heterogeneity within our conduct problem measure, inclusive of the potential for information bias, should be considered in interpreting our results.

Additional limitations include the depth and variety of social risk factors we could consider. First, we used a basic measure of racial discrimination which does not necessarily capture insidious or pervasive forms of structural or implicit racism. Consequently, our results likely underestimate exposure to, and impact of, discrimination. Second, our measure of parental control may conflate harmful coercive parenting with protective parental monitoring, hence should be interpreted with caution. Third we use a simple self-report measure of troublesome friends. Future work should include more complex and ecologically valid peer friendship network approaches. Further, it is likely that additional risk factors (e.g. adverse life-events) and protective factors (e.g. religion), also vary by ethnic group and contribute to differences. For example, despite high exposure to discrimination and parental control, the Indian/Pakistani/Bangladeshi group had fewer conduct problems. This suggests an important avenue for further analyses.

Finally, at present, analyses are cross-sectional; therefore, we cannot make strong conclusions about the direction of effects.

### Prevalence in context

Our estimates of the prevalence of conduct problems are higher than in London sub-samples of national studies, i.e., 16% vs. 2% in *London-wide* estimates from the MHCYP [3] and vs. 4% in the MCS (16% vs 4%)(personal communication). This may reflect biases in the London sub-samples of national studies. For example, the MHCYP and MCS are limited by low participation rates (MCHYP, 52%; MCS-wave 6 [age 14], 61%) [3, 37] and probably under-sampled those from minority and marginalised groups. By comparison, REACH had an 88% participation rate. Studies, like REACH, which were similarly localised – i.e., The Research with East London Adolescents Community Health Survey [38] (RELACHS) and DASH [14] – reported prevalence estimates similar to ours. For example, using the same self-report-SDQ conduct problem measure (“0–3” = low risk, “4–10” = slightly raised to very high risk) RELACHS reported 30·7% (95%CI: 28·2–33·2) of boys had slightly raised to very high risk of conduct problems, almost identical to our estimate 30·6% (95%CI: 28·6–32·6) [38] when scored at the same threshold. Together these data do suggest conduct problems are markedly more common in London schools.

We replicated anticipated associations between social risk factors - racial discrimination, perceived parenting, and troublesome friends - and conduct problems [8–10, 39]. These risk factors are markers of interactive ecological systems, from intimate parent-child interaction to wider friendships in school, all enmeshed within socio-historical context of racism in the UK. Whilst specific mechanisms of action are beyond the scope of the present paper, it is possible each risk factor explored here, though from disparate domains and acting through different developmental pathways, may interactively and cumulatively increase risk of conduct problems. The high prevalence of conduct problems in inner-city schools, alongside strong associations with risk factors for conduct problems, imply clustered social risk factors may be more common in inner-city schools than nationally, and therefore, contribute to a higher prevalence of conduct problems.

Previous localised studies have also found evidence for a second level of inequality - inequality between ethnic groups. Compared with white British peers, greater risks of conduct problems and antisocial behaviour among black Caribbean and mixed white and black ethnicity adolescents have been reported in a 2008 systematic review of ethnic differences and in reports on MCS data [6, 7, 14]. The mean conduct problem scores among both boys and girls in black Caribbean, mixed white and black, and black African groups reported in DASH [40] were also high, and similar to our findings for these groups. The lack of gender differences among black and mixed ethnicity groups found in both REACH and DASH may indicate that the inner-city environment persistently disproportionately impacts black and mixed ethnicity girls. However, for the white British group, mean conduct problem scores were – to a modest extent – higher in DASH than in REACH. For example, mean conduct problem scores among white British boys was 2.72 in DASH (95%CI 2.58–2.85) vs 2.28 in REACH (95%CI 2.09–2.46). For white British girls, the mean conduct problem scores in DASH were 2.30 (95%CI 2.16–2.44) vs REACH 1.79 (95%CI 1.65–2.04). This suggests a possible reduction in prevalence of conduct problems overtime for white British boys and girls, perhaps indicating changes in social risk factors for the white British group, but not all ethnic groups over the last 15 years. Our findings are consistent with one literal manifestation of what has been termed a “white privilege effect”, whereby the white groups were exposed to fewer social risk factors for conduct problems. The greater exposure to these risks among black and mixed groups may push more adolescents along developmental pathways to conduct problems. For example, initial exposure to racial discrimination may cause distress and increase challenging behaviours [18, 39]; parental control may then increase to harsh or coercive control in response; this, in turn, may further increase and entrench conduct problems [41, 42]. As conduct problems develop because of exposure to social risks, peer friendships can exacerbate or mitigate them. In adolescence, typical selection of friends in the same area, often from the same-ethnic group may aggregate or dissipate risk exposures in friendship groups [9]. Peer influence in a friendship group, with either concentrated or diluted risk factors for conduct problems, may amplify ethnic differences in conduct problems. Reduced exposure to the social risks included in the present study, including racial discrimination and troublesome friends, largely accounted for the lower risk of conduct problems among the white British group relative to the rest of the sample.

## Conclusions

Our findings have at least two important implications. First, they suggest conduct problems are markedly more common in inner-city schools, which points to a need for more resources for schools, social care, and health services in diverse urban areas. Second, they suggest variations in conduct problems are, to some extent, rooted in social contexts and experiences, such as experiences of discrimination. This points to the need for interventions – at multiple levels – to prevent conduct problems developing and, in turn, to mitigate associated long-term adverse outcomes. Such strategies, ultimately, may contribute to reducing health and social inequalities among ethnic groups.

## Supplementary Information

**Additional file 1:.** Tables of comparisons between ethnic groups. Description: In the main article, comparisons were made between ethnic groups and the overall sample prevalence in Tables 2,5 and 6. Alternate versions of Tables 2, 5 and 6 in the main article are printed in this additional file using white British as the reference group.

**Additional file 2:.** Descriptive Data Stratified by Gender. Description: The descriptive characteristics presented in Table 1 in the main article are presented for boys and girls separately.

**Additional file 3:.** Sensitivity Analysis. Description: Sensitivity analyses were run to assess the impact of including data from two (originally pilot) schools in specific analyses.

## Data Availability

We welcome and encourage requests from researchers wishing to access REACH data for specific research projects or collaborations. Our data access policy aims to make REACH data as accessible as possible while adhering to legal and ethical principles and protecting the privacy of schools and participants. Our data accesses policy, along with an application form, are available on request from our Data Manager and Project Coordinator at reach@kcl.ac.uk. Further information about REACH and the available data can be found here: https://www.thereachstudy.com/information-for-researchers.html.

## Abbreviations

BAME: black and minority ethnic
CI: Confidence Intervals
DASH: Determinants of Adolescent Social Well-being and Health
DSM: Diagnostic and Statistical Manual
MCS: Millennium Cohort
MHCYP: Mental Health of Children and Young People in England
PBI-S: Modified Parental Bonding Instrument
REACH: Resilience, Ethnicity, and Adolescent Mental Health
REALCOM: Developing multilevel models for REAListically COMplex social science data
RELACHS: The Research with East London Adolescents Community Health Survey
RR: Risk Ratio
SDQ: Strengths and Difficulties Questionnaire

## References

[1] Bevilacqua L, Hale D, Barker ED, Viner R. Conduct problems trajectories and psychosocial outcomes: a systematic review and meta-analysis. Eur Child Adolesc Psychiatr. 2017;27(10):1239–60. 10.1007/s00787-017-1053-4 28983792

[2] Colman I, Murray J, Abbott RA, Maughan B, Kuh D, Croudace TJ, et al. Outcomes of conduct problems in adolescence: 40 year follow-up of national cohort. BMJ. 2009;338. 10.1136/bmj.a2981 PMC2615547 19131382

[3] Dhriti Mandalia KS, Tim Vizard, Tamsin Ford, Anna Goodman, Robert Goodman, Sally McManus. Mental health of children and young people in England, 2017: Behavioural disorders. In: Care DoHaS, editor. NHS Digital. 2018.

[4] Maughan B, Rowe R, Messer J, Goodman R, Meltzer H (2004). Conduct disorder and oppositional defiant disorder in a national sample: developmental epidemiology. J Child Psychol Psychiatry.

[5] Odgers CL, Moffitt TE, Broadbent JM, Dickson N, Hancox RJ, Harrington H (2008). Female and male antisocial trajectories: from childhood origins to adult outcomes. Dev Psychopathol.

[6] Goodman A, Patel V, Leon DA (2008). Child mental health differences amongst ethnic groups in Britain: a systematic review. BMC Public Health.

[7] Zilanawala A, Sacker A, Kelly Y (2018). Mixed ethnicity and behavioural problems in the millennium cohort study. Arch Dis Child.

[8] Maynard MJ, Harding S (2010). Perceived parenting and psychological well-being in UK ethnic minority adolescents. Child Care Health Dev.

[9] McPherson M, Smith-Lovin L, Cook JM (2001). Birds of a feather: Homophily in social networks. Annu Rev Sociol.

[10] Blakemore S-J. Inventing Ourselves The Secret Life of the Teenage Brain: Penguin; 2018 22/03/2018.

[11] Latimer K, Wilson P, Kemp J, Thompson L, Sim F, Gillberg C (2012). Disruptive behaviour disorders: a systematic review of environmental antenatal and early years risk factors. Child Care Health Dev.

[12] Pinquart M (2017). Associations of parenting dimensions and styles with externalizing problems of children and adolescents: an updated meta-analysis. Dev Psychol.

[13] Dishion TJ, Patterson GR. The development and ecology of antisocial behavior in children and adolescents. Developmental psychopathology: Risk, disorder, and adaptation, Vol 3. 2nd ed. London: Wiley; 2006. p. 503–41.

[14] Harding S, Read UM, Molaodi OR, Cassidy A, Maynard MJ, Lenguerrand E (2015). The determinants of young adult social well-being and health (DASH) study: diversity, psychosocial determinants and health. Soc Psychiatry Psychiatr Epidemiol.

[15] Department for Education. Schools, pupils and their characteristics; National tables: SFR28/2017. In: Education Df, editor. Online. 2017.

[16] Brody GH, Chen Y-F, Murry VM, Ge X, Simons RL, Gibbons FX (2006). Perceived discrimination and the adjustment of African American youths: a five-year longitudinal analysis with contextual moderation effects. Child Dev.

[17] Pachter LM, Coll CG (2009). Racism and child health: a review of the literature and future directions. J Dev Behav Pediatr.

[18] Priest N, Paradies Y, Trenerry B, Truong M, Karlsen S, Kelly Y (2013). A systematic review of studies examining the relationship between reported racism and health and wellbeing for children and young people. Soc Sci Med.

[19] Hatch SL, Gazard B, Williams DR, Frissa S, Goodwin L, Hotopf M. Discrimination and common mental disorder among migrant and ethnic groups. Soc Psychiatry Psychiatr Epidemiol. 2016:1–13. 10.1007/s00127-016-1191-x PMC4846681 26875153

[20] Council S. Southwark Joint Strategic Needs Assessment 2018–19: Demography.; 2018.

[21] Lambeth Council. State of the Borough 2016: Lambeth. 2016.

[22] Hatch SL, Woodhead C, Frissa S, Fear NT, Verdecchia M, Stewart R (2012). Importance of thinking locally for mental health: data from cross-sectional surveys representing South East London and England. PloS One.

[23] Knowles GG-A, Beards, SC.; Blakey, R.; Davis, S.; Lowis, K.; Stanyon, D.; Ofori, A.; Turner, A.; Schools Working Group, Pinfold, V.; Bakolis, I.; Reininghaus, U.; Harding, S.; Morgan, C. High Levels of Mental Distress among Young People in Inner-Cities: the Resilience, Ethnicity and AdolesCent Mental Health (REACH) study. Submitted to Journal of Epidemiology and Community Health 2020. 10.1136/jech-2020-214315 PMC8142438 33558428

[24] Southwark Council. Southwark joint strategic needs assessment 2018–19: demography. 2018.

[25] Goodman R (2001). Psychometric properties of the strengths and difficulties questionnaire. J Am Acad Child Adolesc Psychiatry.

[26] Goodman R. Scoring the Strengths & Difficulties Questionnaire for age 4–17 [PDF]. 2014 [Available from: https://www.ehcap.co.uk/content/sites/ehcap/uploads/NewsDocuments/236/SDQEnglishUK4-17scoring-1.PDF.

[27] Muris P, Meesters C, van den Berg F (2003). The strengths and difficulties questionnaire (SDQ)--further evidence for its reliability and validity in a community sample of Dutch children and adolescents. Eur Child Adolesc Psychiatr.

[28] Green H, McGinnity Á, Meltzer H, Ford T, Goodman R. Mental health of children and young people in Great Britain, 2004. 2005.

[29] Goodman R, Ford T, Richards H, Gatward R, Meltzer H (2000). The development and well-being assessment: description and initial validation of an integrated assessment of child and adolescent psychopathology. J Child Psychol Psychiatry.

[30] Office of National Statistics. Key Statistics for Local Authorities in England and Wales 2011 [Available from: http://www.ons.gov.uk/ons/rel/census/2011-census/key-statistics-for-local-authorities-in-england-and-wales/rft-table-ks201ew.xls.

[31] DfE. Free school meals: Guidance for local authorities, maintained schools, academies and free schools. In: Education Df, editor. 2018.

[32] Astell-Burt T, Maynard MJ, Lenguerrand E, Harding S (2012). Racism, ethnic density and psychological well-being through adolescence: evidence from the determinants of adolescent social well-being and health longitudinal study. Ethnicity Health.

[33] University CM. The Common Cold Project: Parental Bonding 2016 [Available from: https://www.cmu.edu/common-cold-project/measures-by-study/psychological-and-social-constructs/childhood-measures/parental-bonding.html.

[34] Parker G, Tupling H, Brown L (1979). A parental bonding instrument. Psychol Psychother Theory Res Pract.

[35] Localio AR, Margolis DJ, Berlin JA (2007). Relative risks and confidence intervals were easily computed indirectly from multivariable logistic regression. J Clin Epidemiol.

[36] Carpenter JR, Goldstein H, Kenward MG. REALCOM-IMPUTE Software for Multilevel Multiple Imputation with Mixed Response Types. J Stat Softw. 2011;45(5).

[37] Studies CfL. Millennium cohort study sixth sweep (MCS6): technical report: UCL Institute of education, 2017. 2017.

[38] Stansfeld S, Haines M, Booy R, Taylor S, Viner R, Head J, et al. The health of young people in East London: the RELACHS study, 2001. 2003.

[39] Evans SZ, Simons LG, Simons RL (2016). Factors that influence trajectories of delinquency throughout adolescence. J Youth Adolesc.

[40] Maynard MJ, Harding S, Minnis H (2007). Psychological well-being in black Caribbean, black African, and white adolescents in the UK Medical Research Council DASH study. Soc Psychiatry Psychiatr Epidemiol.

[41] Burt SA, McGue M, Krueger RF, Iacono WG (2005). How are parent-child conflict and childhood externalizing symptoms related over time? Results from a genetically informative cross-lagged study. Dev Psychopathol.

[42] Micalizzi L, Wang M, Saudino KJ. Difficult temperament and negative parenting in early childhood: a genetically informed cross-lagged analysis. Dev Sci. 2017;20(2). 10.1111/desc.12355. 10.1111/desc.12355 PMC4840089 26490166

