# Correction to: Prevalence of conduct problems and social risk factors in ethnically diverse inner-city schools

**DOI:** 10.1186/s12889-021-12258-7

**Published:** 2021-11-29

**Authors:** Rachel Blakey, Craig Morgan, Charlotte Gayer-Anderson, Sam Davis, Stephanie Beards, Seeromanie Harding, Vanessa Pinfold, Kamaldeep Bhui, Gemma Knowles, Essi Viding

**Affiliations:** 1 Health Service and Population Research Department, Institute of Psychiatry, Psychology and Neuroscience, King’s College London, London, UK; 2 ESRC Centre for Society and Mental Health, Institute of Psychiatry, Psychology and Neuroscience, King’s College London, London, SE5 8AF UK; 3 Department of Nutritional Sciences, School of Life Course Sciences, Faculty of Life Sciences & Medicine, King’s College London, Stand, WC2R 2LS UK; 4 The McPin Foundation, 7-14 Great Dover Street, London, SE1 4YR UK; 5 Centre for Psychiatry, Wolfson Institute of Preventive Medicine, Barts & The London, Blakey et al. BMC Public Health (2021) 21:849 Page 12 of 13 Queen Mary University of London, Charterhouse Square Campus, Old Anatomy Building, London, EC1M 6BQ UK; 6 Developmental Risk and Resilience Unit, University College London, 26 Bedford Way, London, WC1H 0AP UK

**Correction to: BMC Public Health 21, 849 (2021)**

**https://doi.org/10.1186/s12889-021-10834-5**

It was highlighted that in the original article [1] the Acknowledgments section was incomplete. This Correction article shows the correct section. The original article has been updated.

Acknowledgements

We would like to acknowledge all members of our Young Person’s advisory group who have contributed throughout the REACH study. We would also like to acknowledge Alexis Karamanos who prepared comparison data from the Millennium Cohort Study. This paper represents independent research in part supported by the ESRC Centre for Society and Mental Health at King’s College London (ESRC Reference: ES/S012567/1). The views expressed are those of the author(s) and not necessarily those of the ESRC or King’s College London.
