# Supplementary material for: Prevalence of conduct problems and social risk factors in ethnically diverse inner-city schools
Source: BMC Public Health. 2021 May 3;21:849. doi: 10.1186/s12889-021-10834-5 (PMC8091508; doi:10.1186/s12889-021-10834-5)
Supplement: Supplementary file 1 — Additional file 1:. Tables of comparisons between ethnic groups. Description: In the main article, comparisons were made between ethnic groups and the overall sample prevalence in Tables 2,5 and 6. Alternate versions of Tables 2, 5 and 6 in the main article are printed in this additional file using white British as the reference group. [file 12889_2021_10834_MOESM1_ESM.docx]

Additional Table 1· Descriptive Data, Boys Only

|  | *Conduct problems* | | | | **Often Fighting and Bullying* | | | | **Often Rule-breaking* | | | | |
| --- | --- | --- | --- | --- | --- | --- | --- | --- | --- | --- | --- | --- | --- |
|  | ***Low risk*** | ***High risk*** | ***Missing*** | ***χ2*** | ***No*** | ***Perhaps/***  ***Definitely*** | ***Missing*** | ***χ2*** | ***No*** | ***Perhaps/***  ***Definitely*** | ***Missing*** | ***χ2*** |  |
|  | ***n (%)*** | ***n (%)*** | ***n (%)*** |  | ***n (%)*** | ***n (%)*** | ***n (%)*** |  | ***n (%)*** | ***n (%)*** | ***n (%)*** |  |  |
| Total Sample  (2,138) | 1720 (80) | 378 (18) | 40 (2) |  | 1034 (60) | 542 (32) | 134 (8) |  | 925 (54) | 628 (37) | 157 (9) |  |  |
| Year group |  |  |  | 0.23, p = 0.893 |  |  |  | 2·79, p = 0·248 |  |  |  | 20.16 p<0·000 |  |
| 7 | 625 (80) | 142 (18) | 11 (1) |  | 393 (63) | 183 (29) | 52 (8) |  | 374 (60) | 190 (30) | 64 (10) |  |  |
| 8 | 563 (80) | 120 (17) | 18 (3) |  | 314 (58) | 174 (32) | 51 (9) |  | 285 (53) | 201 (37) | 53 (10) |  |  |
| 9 | 532 (81) | 116 (18) | 11 (2) |  | 327 (60) | 185 (34) | 31 (6) |  | 266 (49) | 237 (44) | 40 (7) |  |  |
| Ethnicity |  |  |  | 29.99, p<0.001 |  |  |  | 29.84, p < 0.001 |  |  |  | 25.77 p = 0.002 |  |
| Black African | 442 (81) | 93 (17) | 10 (2) |  | 243 (54) | 163 (36) | 43 (10) |  | 241 (54) | 153 (34) | 55 (12) |  |  |
| Black Caribbean | 262 (75) | 75 (21) | 13 (4) |  | 124 (54) | 80 (35) | 27 (12) |  | 106 (46) | 96 (42) | 29 (13) |  |  |
| Indian, Pakistani, Bangladeshi | 75 (90) | 7 (8) | 1 (1) |  | 41 (64) | 19 (30) | 4 (6) |  | 45 (70) | 16 (25) | 3 (5) |  |  |
| Latin American | 83 (75) | 25 (23) | 2 (2) |  | 39 (51) | 30 (39) | 7 (9) |  | 34 (45) | 30 (39) | 12 (16) |  |  |
| Mixed white and black | 138 (76) | 44 (24) | 0 (0) |  | 88 (59) | 52 (35) | 10 (7) |  | 81 (54) | 58 (39) | 11 (7) |  |  |
| Other black | 38 (75) | 12 (24) | 1 (2) |  | 20 (51) | 16 (41) | 3 (8) |  | 18 (46) | 17 (44) | 4 (10) |  |  |
| Other mixed/multiple | 90 (86) | 15 (14) | 0 (0) |  | 59 (63) | 29 (31) | 5 (5) |  | 38 (41) | 50 (54) | 5 (5) |  |  |
| Other White | 165 (77) | 43 (20) | 5 (2) |  | 107 (67) | 43 (27) | 9 (6) |  | 91 (57) | 57 (36) | 11 (7) |  |  |
| Other/unknown | 130 (80) | 27 (17) | 5 (3) |  | 79 (62) | 35 (27) | 14 (11) |  | 73 (57) | 42 (33) | 13 (10) |  |  |
| White British | 297 (88) | 37 (11) | 3 (1) |  | 234 (73) | 75 (23) | 12 (4) |  | 198 (62) | 109 (34) | 14 (4) |  |  |
| Putative risk factors |  |  |  |  |  |  |  |  |  |  |  |  |  |
| Receives free school meals |  |  |  | 22.46, p< 0.001 |  |  |  | 7.44, p = 0.006 |  |  |  | 4.35, p = 0.037 |  |
| *yes* | 339 (74) | 114 (25) | 8 (2) |  | 163 (54) | 115 (38) | 23 (8) |  | 147 (49) | 125 (42) | 29 (10) |  |  |
| *No* | 1303 (83) | 239 (15) | 21 (1) |  | 817 (62) | 398 (30) | 93 (7) |  | 731 (56) | 469 (36) | 108 (8) |  |  |
| Experienced Racial Discrimination |  |  |  | 42.15, p<0.001 |  |  |  | 56.95, p<0.001 |  |  |  |  |  |
| *yes* | 357 (72) | 133 (27) | 3 (1) |  | 188 (47) | 190 (28) | 31 (3) |  | 170 (43) | 200 (50) | 29 (7) |  |  |
| *No* | 1183 (85) | 195 (14) | 13 (1) |  | 776 (69) | 315 (48) | 21 (5) |  | 703 (63) | 376 (34) | 43 (4) |  |  |
| *Troublesome friends |  |  |  | 97.83, p<0.001 |  |  |  | 112.42, p<0.001 |  |  |  | 87.54, p<0.001 |  |
| *None* | 327 (88) | 40 (11) | 5 (1) |  | 282 (76) | 67 (18) | 23 (6) |  | 258 (69) | 86 (23) | 28 (8) |  |  |
| *A few* | 863 (85) | 148 (15) | 8 (1) |  | 656 (64) | 334 (33) | 29 (3) |  | 584 (57) | 397 (39) | 38 (4) |  |  |
| *Many or all* | 124 (58) | 86 (40) | 3 (1) |  | 75 (35) | 130 (61) | 8 (4) |  | 67 (31) | 130 (61) | 16 (8) |  |  |
| *Parental Control |  |  |  | 6.17, p<0.013 |  |  |  | 16.66, p<0.001 |  |  |  | 1.64, p= 0.200 |  |
| *High* | 443 (79) | 109 (20) | 6 (1) |  | 321 (58) | 212 (38) | 25 (4) |  | 305 (55) | 219 (39) | 34 (6) |  |  |
| *Low* | 576 (85) | 97 (14) | 4 (1) |  | 475 (70) | 190 (28) | 12 (2) |  | 409 (60) | 252 (37) | 16 (2) |  |  |
| *Parental Care |  |  |  | 22.78, p<0.001 |  |  |  | 13.92, p<0.001 |  |  |  | 37.23, p<0.001 |  |
| *High* | 574 (87) | 78 (12) | 5 (1) |  | 455 (69) | 184 (28) | 18 (3) |  | 432 (66) | 199 (30) | 26 (4) |  |  |
| *Low* | 446 (77) | 127 (22) | 5 (1) |  | 341 (59) | 218 (38) | 19 (3) |  | 283 (49) | 271 (47) | 24 (4) |  |  |

_Percentages and n unweighted_

_*Administered at 10 of 12 schools, total sample (n= 3535)._

Additional Table 2· Descriptive Data, Girls Only

|  | *Conduct problems* | | | | **Often Fighting and Bullying* | | | | **Often Rule-breaking* | | | |
| --- | --- | --- | --- | --- | --- | --- | --- | --- | --- | --- | --- | --- |
|  | ***Low risk*** | ***High risk*** | ***Missing*** | ***χ2*** | ***No*** | ***Perhaps/***  ***Definitely*** | ***Missing*** | ***χ2*** | ***No*** | ***Perhaps/***  ***Definitely*** | ***Missing*** | ***χ2*** |
|  | ***n (%)*** | ***n (%)*** | ***n (%)*** |  | ***n (%)*** | ***n (%)*** | ***n (%)*** |  | ***n (%)*** | ***n (%)*** | ***n (%)*** |  |
| Total Sample (2,215) | 1852 (84) | 332 (15) | 31 (1) |  | 1152 (63) | 590 (33) | 83 (5) |  | 1111 (61) | 612 (34) | 102 (5) |  |
| Year group |  |  |  | 0.23, p = 0.893 |  |  |  | 2·79, p = 0·248 |  |  |  | 33.16, p < 0.001 |
| 7 | 590 (84) | 103 (15) | 8 (1) |  | 443 (63) | 215 (31) | 43 (6) |  | 468 (67) | 178 (25) | 55 (8) |  |
| 8 | 506 (85) | 80 (13) | 10 (2) |  | 371 (62) | 198 (33) | 27 (5) |  | 357 (60) | 213(36) | 26 (4) |  |
| 9 | 450 (85) | 71 (13) | 7 (1) |  | 338 (64) | 177 (34) | 13 (2) |  | 286 (54) | 221 (42) | 21 (4) |  |
| Ethnicity |  |  |  | 29.99, p<0.001 |  |  |  | 31.33, p < 0.001 |  |  |  | 47.92, p< 0.001 |
| Black African | 465 (82) | 92 (16) | 11 (2) |  | 280 (59) | 172 (36) | 25 (5) |  | 274 (57) | 168 (35) | 35 (7) |  |
| Black Caribbean | 278 (75) | 84 (23) | 7 (2) |  | 168 (62) | 86 (32) | 18 (7) |  | 148 (54) | 106 (39) | 18 (7) |  |
| Indian, Pakistani, Bangladeshi | 90 (92) | 7 (7) | 1 (1) |  | 65 (73) | 20 (22) | 4 (4) |  | 70 (79) | 15 (17) | 4 (4) |  |
| Latin American | 97 (91) | 10 (9) | 0 (0) |  | 36 (48) | 38 (51) | 1 (1) |  | 35 (47) | 32 (43) | 8 (11) |  |
| Mixed white and black | 158 (80) | 37 (19) | 3 (2) |  | 103 (65) | 47 (30) | 8 (5) |  | 83 (53) | 67 (42) | 8 (5) |  |
| Other black | 63 (83) | 13 (17) | 0 (0) |  | 37 (54) | 28 (41) | 3 (4) |  | 38 (56) | 27 (40) | 3 (4) |  |
| Other mixed/multiple | 105 (80) | 24 (18) | 3 (2) |  | 70 (60) | 37 (32) | 9 (8) |  | 69 (59) | 40 (34) | 7 (6) |  |
| Other White | 180 (92) | 16 (8) | 0 (0) |  | 88 (61) | 49 (34) | 7 (5) |  | 87 (60) | 50 (35) | 7 (5) |  |
| Other/unknown | 115 (82) | 22 (16) | 4 (3) |  | 76 (65) | 36 (31) | 5 (4) |  | 76 (65) | 36 (31) | 5 (4) |  |
| White British | 301 (91) | 27 (8) | 2 (1) |  | 229 (74) | 77 (25) | 3 (1) |  | 231 (75) | 71 (23) | 7 (3) |  |
| Putative risk factors |  |  |  |  |  |  |  |  |  |  |  |  |
| Receives free school meals |  |  |  | 22.46, p< 0.001 |  |  |  | 7.44, p = 0.006 |  |  |  | 1.10, p = 0.293 |
| *yes* | 423 (82) | 83 (16) | 9 (1) |  | 228 (64) | 109 (31) | 19 (5) |  | 207 (58) | 128 (36) | 21 (5) |  |
| *No* | 1326 (84) | 231 (15) | 17 (2) |  | 855 (63) | 446 (33) | 57 (4) |  | 835 (61) | 452 (33) | 71 (5) |  |
| Experienced Racial Discrimination |  |  |  | 42.15, p<0.001 |  |  |  | 56.95, p<0.001 |  |  |  | 41.32, p < 0.001 |
| *yes* | 448 (76) | 139 (24) | 1 (0) |  | 244 (50) | 232 (48) | 11 (2) |  | 248 (51) | 222 (46) | 17 (3) |  |
| *No* | 1301 (87) | 175 (12) | 18 (1) |  | 875 (71) | 326 (27) | 27 (2) |  | 829 (68) | 364 (30) | 35 (3) |  |
| *Troublesome friends |  |  |  | 97.83, p<0.001 |  |  |  | 112.42, p<0.001 |  |  |  | 195.10, p < 0.001 |
| *None* | 629 (93) | 39 (6) | 9 (1) |  | 523 (77) | 137 (20) | 17 (3) |  | 538 (79) | 117 (17) | 22 (3) |  |
| *A few* | 750 (84) | 137 (15) | 4 (0) |  | 563 (63) | 314 (35) | 14 (2) |  | 504 (57) | 360 (40) | 27 (3) |  |
| *Many or all* | 113 (62) | 65 (36) | 4 (2) |  | 47 (26) | 125 (69) | 10 (5) |  | 50 (27) | 120 (67) | 12 (7) |  |
| *Parental Control |  |  |  | 6.17, p<0.013 |  |  |  | 16.66, p<0.001 |  |  |  | 17.45, p < 0.001 |
| *High* | 563 (80) | 131 (19) | 8 (1) |  | 429 (61) | 254 (36) | 19 (3) |  | 397 (57) | 276 (39) | 29 (4) |  |
| *Low* | 685 (89) | 77 (10) | 6 (1) |  | 527 (69) | 230 (30) | 11 (1) |  | 524 (68) | 229 (30) | 15 (2) |  |
| *Parental Care |  |  |  | 22.78, p<0.001 |  |  |  | 13.92, p<0.001 |  |  |  | 64.60 p < 0.001 |
| *High* | 679 (90) | 66 (9) | 10 (1) |  | 547 (72) | 190 (25) | 18 (2) |  | 546 (72) | 186 (25) | 23 (3) |  |
| *Low* | 570 (80) | 141 (20) | 5 (1) |  | 411 (57) | 293 (41) | 12 (2) |  | 377 (53) | 318 (44) | 21 (3) |  |

_Percentages and n unweighted_

_*Administered at 10 of 12 schools, total sample (n= 3535)._
