# Supplementary material for: Prevalence of conduct problems and social risk factors in ethnically diverse inner-city schools
Source: BMC Public Health. 2021 May 3;21:849. doi: 10.1186/s12889-021-10834-5 (PMC8091508; doi:10.1186/s12889-021-10834-5)
Supplement: Supplementary file 2 — Additional file 2:. Descriptive Data Stratified by Gender. Description: The descriptive characteristics presented in Table 1 in the main article are presented for boys and girls separately. [file 12889_2021_10834_MOESM2_ESM.docx]

**Sensitivity Analyses, 10 schools n = 3535**

|  | Risk of conduct problems | |
| --- | --- | --- |
|  | **Unadjusted RR (95%CI)** | **Adjusted RR (95%CI)** |
| Ethnicity |  |  |
| Black African | 1.00 (0.85 - 1.18) | 1.00 (0.85 - 1.18) |
| Black Caribbean | 1.37 (1.12 - 1.65) | 1.37 (1.13 - 1.65) |
| Indian/Pakistani/Bangladeshi | 0.49 (0.25 - 0.85) | 0.49 (0.25 - 0.85) |
| Latin American | 1.03 (0.66 - 1.51) | 1.07 (0.70 - 1.57) |
| Mixed white and black | 1.29 (0.99 - 1.64) | 1.27 (0.98 - 1.63) |
| Other black | 1.33 (0.84 - 2.00) | 1.34 (0.85 - 2.01) |
| Other mixed/multiple | 1.04 (0.72 - 1.44) | 1.04 (0.73 - 1.45) |
| Other white | 0.84 (0.60 - 1.14) | 0.86 (0.62 - 1.17) |
| Other/unknown | 1.11 (0.81 - 1.49) | 1.09 (0.79 - 1.47) |
| White British | 0.64 (0.49 - 0.81) | 0.66 (0.51 -0.84) |

Additional Table 3. Risk Ratios for conduct problems by ethnic group

*_RR = Risk ratio, 95%CI = 95% Confidence Interval_*

*_Reference group is overall prevalence_*

*_Adjusted for clustering by school, year group, gender, and free school meals_*

|  | *Raised to high risk of conduct problems* | |
| --- | --- | --- |
|  | ***Unadjusted RR (95%CI)*** | ***Adjusted RR (95%CI)*** |
| Overall gender difference |  |  |
| Boy | 1 | 1 |
| Girl | 0.76 (0.68 - 0.84) | 0.74 (0.64 - 0.83) |
| Within ethnic group |  |  |
| Black African | 0.91 (0.71 - 1.11) | 0.91 (0.71 - 1.11) |
| Black Caribbean | 1.01 (0.63 - 1.39) | 0.97 (0.63 - 1.32) |
| Indian/Pakistani/Bangladeshi | 0.93 (-0.42 - 2.27) | 1.37 (-1.70 - 4.45) |
| Latin American | 0.41 (0.06 - 0.75) | 0.41 (0.06 - 0.76) |
| Mixed white and black | 0.79 (0.50 - 1.08) | 0.78 (0.46 - 1.10) |
| Other black | 0.82 (0.24 - 1.39) | 0.83 (0.27 - 1.40) |
| Other mixed/multiple | 1.06 (0.58 - 1.53) | 1.01 (0.49 - 1.54) |
| Other white | 0.30 (-0.04 - 0.64) | 0.29 (-0.04 - 0.63) |
| Other/unknown | 0.84 (0.11 - 1.58) | 0.86 (0.10 - 1.61) |
| White British | 0.74 (0.27 - 1.21) | 0.71 (0.20 - 1.22) |

Additional Table 4. Risk Ratios for conduct problems by gender within ethnic group

*_RR = Risk ratio, 95%CI = 95% Confidence Interval_*

*_Adjusted for clustering by school, year group, and free school meals_*

Additional Table 5. Risk Ratios for conduct problems by putative risk factors

|  | *Risk of conduct problems* | |
| --- | --- | --- |
|  | ***Unadjusted RR (95%CI)*** | ***Adjusted RR (95%CI)*** |
| Receives free school meals | 1.45 (1.06 - 1.84) | 1.42 (1.00 - 1.84) |
| Experienced Racial Discrimination | 1.96 (1.51 - 2.41) | 2.00 (1.57 - 2.43) |

*_RR = Risk ratio, 95%CI = 95% Confidence Interval_*

*_Adjusted for clustering by school, year group, and free school meals_*

Additional Table 6 Risk Ratios for putative risk factors by ethnic group

|  | Receives free school meals | Experienced Racial Discrimination |
| --- | --- | --- |
|  | % (95%CI) | % (95%CI) |
| Overall prevalence in sample | 0.20 (0.16 - 0.24) | 0.28 (0.23 - 0.33) |
| *Ethnicity* | RR (95%CI) | RR (95%CI) |
| Black African | 1.04 (0.90 - 1.20) | 1.12 (1.00 - 1.26) |
| Black Caribbean | 1.14 (0.94 - 1.37) | 1.05 (0.89 - 1.23) |
| Indian/Pakistani/Bangladeshi | 0.71 (0.45 - 1.08) | 1.20 (0.89 - 1.57) |
| Latin American | 0.72 (0.45 - 1.09) | 1.05 (0.76 - 1.40) |
| Mixed white and black | 1.40 (1.12 - 1.72) | 1.06 (0.85 - 1.30) |
| Other black | 0.88 (0.53 - 1.37) | 0.87 (0.57 - 1.28) |
| Other mixed/multiple | 1.18 (0.88 - 1.56) | 1.34 (1.06 - 1.67) |
| Other white | 0.64 (0.45 - 0.87) | 0.77 (0.59 - 0.98) |
| Other/unknown | 1.35 (1.05 - 1.72) | 1.16 (0.92 - 1.44) |
| White British | 0.80 (0.65 - 0.97) | 0.60 (0.49 -0.73) |

*_RR = Risk ratio, 95%CI = 95% Confidence Interval_*

*_Reference group is the overall prevalence_*

*_Adjusted for clustering by school._*
