# Supplementary material for: Prevalence of conduct problems and social risk factors in ethnically diverse inner-city schools
Source: BMC Public Health. 2021 May 3;21:849. doi: 10.1186/s12889-021-10834-5 (PMC8091508; doi:10.1186/s12889-021-10834-5)
Supplement: Supplementary file 3 — Additional file 3:. Sensitivity Analysis. Description: Sensitivity analyses were run to assess the impact of including data from two (originally pilot) schools in specific analyses. [file 12889_2021_10834_MOESM3_ESM.pdf]

Alternate Table 2. Risk Ratios for conduct problems by ethnic group (white British as reference group)

	**Conduct problems**		***Fighting/Bullying**		***Rule-breaking**	
	**Unadjusted RR (95%CI)**	**Adjusted RR (95%CI)**	**Unadjusted RR (95%CI)**	**Adjusted RR (95%CI)**	**Unadjusted RR (95%CI)**	**Adjusted RR (95%CI)**
**Ethnicity**						
**Black African**	1.56 (1.13, 1.99)	1.53 (1.10, 1.96)	1.57 (1.40, 1.74)	1.60 (1.38, 1.82)	1.24 (1.01, 1.47)	1.27 (1.02, 1.52)
**Black Caribbean**	2.22 (1.53, 2.90)	2.16 (1.50, 2.83)	1.58 (1.39, 1.77)	1.60 (1.41, 1.79)	1.48 (1.20, 1.76)	1.51 (1.22, 1.81)
**Indian/Pakistani/Bangladeshi**	0.83 (0.25, 1.40)	0.82 (0.27, 1.36)	1.27 (0.84, 1.69)	1.29 (0.84, 1.75)	0.76 (0.49, 1.04)	0.76 (0.49, 1.04)
**Latin American**	1.48 (0.87, 2.09)	1.49 (0.90, 2.08)	1.88 (1.54, 2.22)	1.92 (1.54, 2.30)	1.57 (1.29, 1.86)	1.61 (1.31, 1.91)
**Mixed white and black**	2.09 (1.56, 2.62)	2.01 (1.50, 2.52)	1.42 (1.18, 1.66)	1.43 (1.17, 1.70)	1.42 (1.11, 1.74)	1.46 (1.11, 1.80)
**Other black**	1.66 (0.96, 2.35)	1.63 (0.94, 2.31)	1.47 (1.21, 1.73)	1.48 (1.21, 1.75)	1.59 (1.30, 1.87)	1.67 (1.35, 1.98)
**Other mixed/multiple**	1.61 (1.12, 2.10)	1.55 (1.08, 2.02)	1.31 (1.09, 1.53)	1.32 (1.07, 1.56)	1.15 (0.94, 1.35)	1.13 (0.92, 1.35)
**Other white**	1.92 (1.25, 2.58)	1.90 (1.25, 2.56)	1.82 (1.48, 2.16)	1.88 (1.48, 2.27)	1.52 (1.13, 1.91)	1.63 (1.23, 2.02)
**Other/unknown**	1.37 (0.87, 1.86)	1.38 (0.88, 1.87)	1.30 (1.07, 1.52)	1.31 (1.08, 1.54)	1.25 (1.00, 1.49)	1.28 (1.01, 1.55)
**White British**	1	1	1	1	1	1

RR = Risk ratio, 95%CI = 95% Confidence Interval
Reference group is white British
Adjusted for clustering by school, year group, gender, and free school meals
**Administered at 10 of 12 schools, total sample (n= 3535).*

Alternate Table 5 Risk ratios for putative risk factors by ethnic group (white British as reference group)

	**Receives free school meals**	**Experienced Racial Discrimination**	***A few to many or all troublesome friend's vs none**	***Parental High Control**	***Parental Low Care**
**Ethnicity**	RR (95%CI)	RR (95%CI)	RR (95%CI)	RR (95%CI)	RR (95%CI)
**Black African**	1.25 (0.81, 1.25)	1.88 (1.22, 2.54)	1.14 (1.05, 1.22)	1.74 (1.39, 2.09)	1.08 (0.90, 1.27)
**Black Caribbean**	1.36 (0.92, 1.36)	1.61 (0.95, 2.27)	1.22 (1.12, 1.32)	1.76 (1.38, 2.14)	1.03 (0.87, 1.19)
**Indian/Pakistani/Bangladeshi**	0.98 (0.32, 0.98)	2.02 (1.06, 2.98)	1.05 (0.91, 1.18)	1.73 (1.29, 2.16)	0.92 (0.73, 1.12)
**Latin American**	0.85 (0.49, 0.85)	1.66 (1.10, 2.22)	1.07 (0.93, 1.21)	2.00 (1.49, 2.51)	1.17 (1.00, 1.35)
**Mixed white and black**	1.67 (1.20, 1.67)	1.70 (1.17, 2.24)	1.21 (1.05, 1.38)	1.50 (1.18, 1.82)	1.02 (0.85, 1.19)
**Other black**	1.10 (0.69, 1.10)	1.58 (0.75, 2.42)	1.09 (0.93, 1.24)	1.64 (1.23, 2.05)	0.89 (0.71, 1.08)
**Other mixed/multiple**	1.40 (0.97, 1.40)	2.32 (1.39, 3.24)	1.08 (0.95, 1.21)	1.42 (1.08, 1.76)	1.06 (0.80, 1.31)
**Other white**	0.80 (0.51, 0.80)	1.33 (1.00, 1.67)	1.05 (0.88, 1.22)	1.34 (1.05, 1.63)	0.89 (0.75, 1.04)
**Other/unknown**	1.46 (0.80, 1.46)	1.98 (1.24, 2.72)	1.08 (0.95, 1.21)	1.64 (1.27, 2.00)	1.08 (0.87, 1.28)
**White British**	1	1	1	1	1

RR = Risk ratio, 95%CI = 95% Confidence Interval
Reference group is white British
Adjusted for clustering by school, year group, gender, and free school meals
**Administered at 10 of 12 schools, total sample (n= 3535).*

Alternate Table 6. Risk ratios for conduct problems by ethnic group accounting for putative risk factors (white British as reference group)

	Base model: age, gender, free school meals	Model 1: base model + racial discrimination	Model 2: model 1+ friends who get into trouble	Model 3: model 2 + high perceived over protection	Model 4: model 3 + low perceived parental care
	RR (95%CI)	RR (95%CI)	RR (95%CI)	RR (95%CI)	RR (95%CI)
**Conduct problems**					
**Black African**	1.53 (1.06, 1.99)	1.39 (0.95, 1.84)	1.28 (0.85, 1.72)	1.21 (0.79, 1.64)	1.19 (0.77, 1.60)
**Black Caribbean**	2.11 (1.42, 2.79)	1.99 (1.34, 2.64)	1.81 (1.14, 2.49)	1.72 (1.06, 2.37)	1.75 (1.07, 2.44)
**Indian/Pakistani/Bangladeshi**	0.75 (0.24, 1.26)	0.64 (0.21, 1.07)	0.62 (0.20, 1.03)	0.58 (0.20, 0.97)	0.59 (0.19, 0.99)
**Latin American**	1.62 (1.09, 2.16)	1.50 (0.95, 2.05)	1.42 (0.82, 2.02)	1.31 (0.74, 1.87)	1.27 (0.72, 1.82)
**Mixed white and black**	1.93 (1.43, 2.43)	1.81 (1.31, 2.31)	1.71 (1.25, 2.16)	1.66 (1.23, 2.09)	1.68 (1.20, 2.17)
**Other black**	2.04 (1.33, 2.75)	1.97 (1.17, 2.77)	1.95 (1.08, 2.83)	1.89 (1.04, 2.75)	2.01 (1.08, 2.94)
**Other mixed/multiple**	1.56 (0.84, 2.29)	1.36 (0.65, 2.07)	1.27 (0.58, 1.96)	1.24 (0.55, 1.93)	1.22 (0.53, 1.91)
**Other white**	1.31 (0.76, 1.87)	1.28 (0.73, 1.82)	1.23 (0.66, 1.80)	1.20 (0.63, 1.78)	1.25 (0.64, 1.87)
**Other/unknown**	1.66 (1.12, 2.20)	1.50 (0.89, 2.10)	1.48 (0.77, 2.20)	1.40 (0.71, 2.09)	1.40 (0.71, 2.08)
**White British**	1	1	1	1	1
**Fighting/Bullying behaviour**					
**Black African**	1.58 (1.36, 1.80)	1.49 (1.25, 1.72)	1.44 (1.18, 1.71)	1.41 (1.15, 1.66)	1.40 (1.14, 1.66)
**Black Caribbean**	1.58 (1.39, 1.77)	1.51 (1.27, 1.74)	1.38 (1.10, 1.66)	1.34 (1.06, 1.62)	1.35 (1.07, 1.62)
**Indian/Pakistani/Bangladeshi**	1.28 (0.81, 1.74)	1.15 (0.69, 1.60)	1.15 (0.66, 1.65)	1.12 (0.63, 1.62)	1.14 (0.60, 1.67)
**Latin American**	1.90 (1.55, 2.26)	1.86 (1.44, 2.27)	1.93 (1.40, 2.46)	1.88 (1.34, 2.41)	1.88 (1.34, 2.42)
**Mixed white and black**	1.41 (1.15, 1.67)	1.33 (1.08, 1.58)	1.23 (0.96, 1.49)	1.21 (0.95, 1.46)	1.21 (0.94, 1.48)
**Other black**	1.84 (1.45, 2.24)	1.83 (1.31, 2.34)	1.92 (1.24, 2.61)	1.90 (1.22, 2.57)	1.97 (1.24, 2.69)
**Other mixed/multiple**	1.47 (1.21, 1.73)	1.32 (1.05, 1.60)	1.28 (0.96, 1.60)	1.27 (0.95, 1.59)	1.26 (0.92, 1.60)
**Other white**	1.31 (1.07, 1.55)	1.29 (1.05, 1.53)	1.27 (0.99, 1.55)	1.26 (0.98, 1.54)	1.29 (0.99, 1.59)
**Other/unknown**	1.30 (1.06, 1.54)	1.19 (0.97, 1.40)	1.16 (0.88, 1.44)	1.13 (0.86, 1.40)	1.12 (0.84, 1.39)
**White British**	1	1	1	1	1
**Rule-breaking behaviour**					
**Black African**	1.26 (1.01, 1.52)	1.18 (0.91, 1.46)	1.10 (0.79, 1.41)	1.07 (0.76, 1.38)	1.05 (0.75, 1.35)

**Black Caribbean**	1.51 (1.21, 1.81)	1.45 (1.11, 1.80)	1.36 (0.95, 1.76)	1.32 (0.91, 1.73)	1.34 (0.90, 1.78)
**Indian/Pakistani/Bangladeshi**	0.75 (0.49, 1.02)	0.67 (0.40, 0.93)	0.61 (0.35, 0.87)	0.59 (0.33, 0.85)	0.59 (0.31, 0.87)
**Latin American**	1.61 (1.32, 1.91)	1.56 (2.04, 2.76)	1.60 (1.17, 2.04)	1.55 (1.12, 1.99)	1.55 (1.10, 1.99)
**Mixed white and black**	1.45 (1.11, 1.80)	1.40 (1.04, 1.76)	1.33 (0.90, 1.76)	1.31 (0.88, 1.74)	1.32 (0.86, 1.78)
**Other black**	1.62 (1.22, 2.02)	1.59 (1.12, 2.06)	1.68 (0.99, 2.36)	1.65 (0.96, 2.34)	1.73 (0.98, 2.48)
**Other mixed/multiple**	1.66 (1.35, 1.97)	1.56 (1.23, 1.89)	1.64 (1.20, 2.07)	1.62 (1.18, 2.07)	1.65 (1.15, 2.16)
**Other white**	1.27 (0.99, 1.56)	1.26 (0.95, 1.56)	1.26 (0.92, 1.60)	1.25 (0.91, 1.58)	1.30 (0.94, 1.66)
**Other/unknown**	1.12 (0.91, 1.34)	1.04 (0.82, 1.25)	1.00 (0.73, 1.27)	0.97 (0.71, 1.23)	0.95 (0.70, 1.20)
**White British**	1	1	1	1	1

RR = Risk ratio, 95%CI = 95% Confidence Interval

Reference group is white British in each model

For the purpose of comparison across models, all models include observations from 10 schools n =3535
